# An update comprehensive review on the effects of transcutaneous electrical nerve stimulation for postnatal physical and psychological disorders

**DOI:** 10.3389/fneur.2025.1594422

**Published:** 2025-06-25

**Authors:** Beibei Chen, Chunyan Chen, Xiumin Zhao, Yan Xu

**Affiliations:** ^1^Hangzhou Women’s Hospital (Hangzhou Maternity and Child Health Care Hospital), Hangzhou, Zhejiang, China; ^2^Department of Nephrology, Jiaxing Hospital of Traditional Chinese Medicine, Jiaxing, Zhejiang, China; ^3^Department of Obstetrics and Gynecology, Taizhou Municipal Hospital (Taizhou University Affiliated Municipal Hospital), School of Medicine, Taizhou University, Taizhou, Zhejiang, China

**Keywords:** transcutaneous electrical nerve stimulation, postnatal rehabilitation, pelvic floor dysfunction, treatment, psychological disorders

## Abstract

Postnatal rehabilitation is crucial to women’s physical and mental health, not only to the quality of life of the mother herself, but also to the well-being of the whole family. Transcutaneous Electrical Nerve Stimulation (TENS) has been gradually attracting attention as an emerging means of postnatal rehabilitation. We summarized the current evidence related to this topic by a comprehensive review. Relevant studies demonstrated that TENS is effective for treating postnatal rehabilitation. TENS showed a good analgesia without any severe adverse effects with a frequency of 100 Hz with a pulse duration of 100 μs. Pain significantly decreased and comfort increased after having a cesarean delivery in TENS group compared to the control group. TENS can be applied in the treatment of various postnatal disorders, such as musculoskeletal pain, pelvic floor dysfunction (i.e., postoperative urinary retention, fecal incontinence, and pelvic organ prolapse), sexual dysfunction, sleep disorders, and postpartum depression. Postnatal pain, pelvic floor dysfunction, and sexual dysfunction have been found to associated with the promotion of the pro-inflammatory cytokines and the inhibition of immune cell activity. The mechanisms underlying the protective effects of TENS are modulation of inflammatory responses and immune functions. Thus, TENS is a versatile tool in postpartum rehabilitation, addressing physical and psychological sequelae of childbirth. In future, more large-sample multiple RCTs are still warranted to confirm these findings.

## 1 Introduction

Postnatal rehabilitation is crucial to women’s physical and mental health, not only to the quality of life of the mother herself, but also to the well-being of the whole family (1). Postpartum women face many physical and psychological challenges. Physiologically, the process of labor and delivery causes great trauma to a woman’s body. The pelvic floor muscles are stretched and damaged to varying degrees during labor, which may lead to problems such as urinary incontinence and pelvic organ prolapse (2). In addition, postpartum pain is a common nuisance, including abdominal incision pain and perineal pain. Psychologically, the incidence of postpartum depression should not be underestimated, and new moms may experience low mood, anxiety, insomnia and other symptoms. Traditional methods of postpartum rehabilitation mainly include postpartum exercises, dietary adjustments and psychological counseling. However, these methods may have certain limitations in their implementation. For example, postpartum exercise requires the mother to have enough time and energy and to master the correct method, otherwise it may be counterproductive. Dietary adjustments are often difficult to meet individualized needs. Other treatments, like psychological counseling, requires professional personnel and the effect varies individually (3).

Transcutaneous electrical nerve stimulation (TENS), a specific low-frequency pulse current inputted into the body through the skin to stimulate the nerves to achieve the purpose of treatment and rehabilitation, has been gradually attracting attention as an emerging means of rehabilitation (4). Electrical stimulation technology is to stimulate local vasculature, smooth muscle and innervating nerves through different types of electric currents to promote blood microcirculation, intervene in the patient’s vascular system homeostasis and various systems of the body, in order to achieve the purpose of treating the disease or used for auxiliary treatment.

TENS offers a number of advantages. First, it is a non-invasive treatment that avoids the risks and pain associated with surgery. Secondly, TENS can be customized according to the patient’s specific situation, including stimulation intensity, frequency and time. Furthermore, TENS is easy to operate, and patients can perform the treatment at home under the guidance of a doctor, which improves the compliance of the treatment. International regulatory and practice differences are essential for contextualizing TENS applications, particularly in postnatal care. For example, in Japan, TENS is widely used as a non-prescription, home-based device for conditions like chronic lower back pain, reflecting its accessibility and acceptance in self-management strategies. In China, it emphasizes structured protocols under professional supervision, indicating that TENS is typically administered by healthcare providers in clinical settings. In Europe, TENS is often integrated into multidisciplinary rehabilitation programs, where physiotherapists play a key role in prescribing and adjusting stimulation parameters. In recent years, more and more studies have begun to focus on the application of TENS in postpartum rehabilitation. Studies have shown that TENS can effectively relieve postpartum pain, such as perineal lateral incision pain and cesarean section incision pain. Meanwhile, TENS also has a positive effect on the rehabilitation of pelvic floor muscles, which can enhance the contraction force of pelvic floor muscles and prevent urinary incontinence and pelvic organ prolapse. In addition, TENS may also have a certain auxiliary therapeutic effect on postpartum depression, by stimulating the nerves, regulating the neurotransmitters in the brain, and improving the patient’s emotional state (5).

TENS has a broad application prospect in postpartum rehabilitation. In-depth research on the mechanism of TENS in postpartum rehabilitation, the optimal treatment program and the joint application with other rehabilitation methods are of great significance to improve the quality of life of postpartum women (4). We summarized the current evidence related to this topic by a comprehensive review.

## 2 TENS in postpartum recovery

### 2.1 Pain modulation

Postpartum Pain (PP) refers to a wide range of physiological or pathological pain symptoms that women experience after giving birth, usually triggered by birth trauma, uterine regrowth, hormonal changes and breastfeeding (6). The types of PP include acute pain (e.g., incisional pain, contraction pain, and breast tenderness) and chronic pain (e.g., pelvic floor dysfunction or pubic symphysis separation) (7). The physiological adaptations occurring during gestation and post-cesarean, which induce somatic and/or visceral pain (8). Post-cesarean section (CS) women experience greater functional limitations (mobility/hygiene) than vaginal birth counterparts due to perioperative complications and pain. Postpartum pain duration/severity depends on delivery mode (vaginal vs. CS), parity, breastfeeding patterns, and individual factors (pain thresholds, medical history, cultural context). Acute pain resolves in 7–14 days; chronic pelvic dysfunction may persist >6 months, with CS showing longer recovery. Postpartum pain management employs multimodal protocols: Non-pharmacological first-line therapies (thermotherapy, postural optimization, TENS) address physiological dysfunction, while NSAIDs serve as primary analgesics with opioids restricted to ≤72 h use. Psychological interventions modulate pain processing via corticolimbic pathways. However, the above common therapies are not effective in a substantial proportion of post-pregnancy women.

TENS operates through gate control theory and endogenous opioid release, effectively managing postpartum pain (e.g., perineal tears, cesarean incisions) (9). Studies suggest that TENS reduces pain scores by 40–80% in postpartum women, with minimal side effects (10). Anna Pilch et al. (11) reported that the TENS intervention group demonstrated significant pain reduction post-treatment versus controls (*p* = 0.002). Notably during early ambulation phases, the TENS cohort exhibited minimal pain exacerbation (*p* < 0.001 vs. control group). In line with this study, Özen et al. (12) also demonstrated that the postpartum pain dramatically declined and comfort increased after having a cesarean delivery in TENS group compared to the control group (*p* < 0.05). A previous study also showed that the Numerical Pain Rating Scale (NPRS) was significant decreased in patients who following lower segment cesarean section and underwent TENS treatment as compared the control group (*p* < 0.001). These results suggest that administration of TENS indicates an improvement in pain intensity.

Severe postpartum pain and impaired wound healing are conventionally managed with systemic analgesics (oral medications, Patient-Controlled Analgesia pumps), yet these carry risks of allergic reactions and gastrointestinal dysfunction. NSAID-related complications (e.g., gastrointestinal mucosal injury) further limit therapeutic options, particularly for neuropathic pain originating from peripheral nerve irritation (13). The effect of these drugs on the pain caused by the stimulation of the peri-incision somatic nerves is poor. In recent years, electrical stimulation has achieved excellent results in postpartum pain analgesic treatment, but there is no uniform standard on how to choose the appropriate body surface treatment position (11). In a previous study, TENS application demonstrated statistically significant analgesia (pre-intervention: 7.09 ± 1.2 vs. post-intervention: 6.74 ± 0.9, *p* < 0.05) (10). Three-fourths of parturients (78.0%, 199/255) reported satisfactory early labor pain control with the modality, with high therapeutic endorsement (recommendation rate: 80.4%, 203/255; treatment continuation rate: 71.8%, 183/255). Some investigators compared the effects of sequential interval electrical stimulation at a frequency of 2–100 Hz, electrode placement next to the incision and the classical acupuncture points in Chinese medicine theory on the pain scores and analgesic consumption of patients with mid-abdominal incision surgery. The results showed that the methods of placement can be achieved similar efficacy, and can be used for postpartum incisional pain control (14). This may be related to the increase in local blood circulation and restoration of homeostatic balance of arteriovenous and lymphatic circulation. A cellular experiment demonstrated at the molecular level that electrical stimulation can effectively induce angiogenesis by up-regulating chemokine receptors CXCR4 and CXCR2 in endothelial cells, guiding cell migration and directing cell division, which provides a new therapeutic idea for patients with local angiogenic abnormalities and poor healing in the clinic.

Chronic pelvic pain occurs frequently in primiparous women after for various reasons (9, 15). The causes of inflammation-related pain include: (1). damage to the pain nerve tissue caused by surgery; (2). increased tension in the pelvic and abdominal tissues that continuously pulls and stimulates the sensory nerves; (3). pelvic adhesions. For pelvic inflammation and chronic pelvic pain occurring after pelvic surgery, there is no effective targeted treatment, and a comprehensive treatment model combining different treatment methods is often used in clinical practice (16). Zakariaee et al. (15) found a significant difference between the mean pain severity of the TENS group and that of the group with walking activities. A case report showed that a patient underwent TENS from the first trimester until half-year postpartum, achieving a satisfied analgesia without any adverse effects for the mother (17). A previous RCT also demonstrated that postpartum women received TENS treatment had significant pain reduction in comparison to the control group (*p* < 0.01) (16). Consistently, the patients in TENS Group had statistically lower VAS and VNS scores than the patients in the control Group 1 (*p* < 0.001) (18). In Barbara Dionisi et al.’s study (18), the therapeutic cohort demonstrated an 84.5% dyspareunia amelioration rate following a brief five-session TENS regimen, achieving complete symptom resolution in 95% of participants during the post-intervention phase. Longitudinal assessment at 8-month post-treatment evaluation revealed 100% maintenance of asymptomatic status across the study population. Peng et al. (19) reported that the percentage of VAS score decreased by > 25% after TENS treatment. As a novel and non-invasive approach, TENS has been found to be an effective method for analgesia in labor (19). In the aspect of the nerve stimulation intensity, Olsén et al. (20) found that high-intensity TENS demonstrated superior analgesic efficacy, with a median VAS reduction of Δ49 mm (95%CI: −66.5 to −33.2; VAS 0-100 mm scale), significantly exceeding low-intensity TENS (Δ21 mm, 95%CI: −39.0 to −20.0). Intergroup comparative analysis revealed a clinically meaningful 28-mm differential response (95%CI: 14.0–53.0), establishing high-intensity-TENS as the optimal neuromodulation parameter for sustained pain relief.

Based on the above evidence, TENS may serve as a versatile tool for postpartum pain management, combining neurophysiological action with practical benefits. While its efficacy varies, its safety and adaptability make it a valuable adjunct to conventional therapies, particularly for women prioritizing non-pharmacological options. Further high-quality trials are needed to standardize protocols and expand applications.

### 2.2 Improved pelvic floor dysfunction

Pelvic floor dysfunction disease refers to a class of diseases related to the weakening of the strength of female pelvic floor support structures to varying degrees. The most common cause is the damage to the pelvic floor muscles and connective tissues caused by the process of pregnancy and childbirth (21). These patients are prone to urinary and fecal incontinence, pelvic organ prolapse, vaginal laxity and other symptoms, resulting in reduced sexual activity, seriously affecting the quality of life and physical and mental health of patients. At present, the proportion of middle-aged and elderly women suffering from pelvic floor dysfunction disease in China is as high as 35.3% (22). As time progresses, the symptoms of the disease will gradually worsen, and failure to take timely and effective treatment measures will lead to a further increase in medical costs. Pelvic floor repair includes surgical and non-surgical treatments. Surgical treatments have better cure rates than non-surgical treatments, but they cause anatomical disruption, are prone to recurrence, and have certain risks associated with surgery that should not be ignored. Non-surgical treatment has gradually attracted people’s attention, such as electrical stimulation therapy, biofeedback and Kegel training, etc.

Therapeutic electrical stimulation selectively activates birth-traumatized pelvic floor musculature (puborectalis, iliococcygeus) through depolarization of denervated motor endplates, thereby restoring volitional contractility. Through parametric optimization (20–50 Hz), TENS induces differential neuromuscular responses: higher-frequency modulation (25–50 Hz) elicits phasic contractions in fast-twitch type II fibers critical for urethral sphincter competency, whereas low-frequency stimulation (1–10 Hz) promotes tonic endurance training of slow-twitch type I fibers essential for pelvic organ support. This frequency-specific neuromodulation enables targeted rehabilitation of discrete fascial layers within the endopelvic fascia. TENS stimulation of the sacral nerve region (S2–S4) regulates the bladder-pelvic floor nerve circuit, inhibits overactivity of the urethral muscles, and relieves urgency and frequency of urination.

In clinical application, a variety of modalities are often used in combined treatment, which has achieved better efficacy (23). TENS has been found to be effective in treating urinary incontinence and fecal incontinence. In a previous study, 93 female patients with mild to moderate stress urinary incontinence were treated with TENS combined with biofeedback pelvic floor rehabilitation therapy, placing electrodes inside the vagina to form a low-frequency impulse electrical stimulation with a frequency of 20–60 Hz, and collecting the electromyographic signals generated during the contraction of the pelvic floor muscles through the electrodes for feedback to the patients. The results showed that the patients’ pelvic floor electromyographic parameters and questionnaire scores improved significantly (24). It was showed that the combination of biofeedback and electrical stimulation with Kegel training can improve the pelvic floor muscle strength of patients and achieve better therapeutic effects. For female patients with idiopathic urgency urinary incontinence refractory to pharmacological interventions, both pudendal nerve stimulation and transvaginal electrical stimulation modalities exhibit significantly high effectiveness (25). Comparative clinical studies reveal that low-frequency electrostimulation achieves 38% greater therapeutic success rates than pharmacological/physical interventions for peripartum and postsurgical urinary retention, concurrently decreasing mean residual urine volume by 52 ± 6 mL. The observed efficacy correlates with bioelectrically modulated improvement in detrusor-sphincter synergy, as quantified by urodynamic pressure-flow studies (26). Chew et al. demonstrated that S3 TENS is a promising noninvasive therapy to treat fecal incontinence (27). They found that the fecal incontinence severity index (FISI) improved in nearly 70% after 3 months of TENS treatment (*p* < 0.01), and all components of fecal incontinence quality of life scale (FIQOL) improved. In addition, 15 (88%) patients scored ≥ 6/10 for bowel control. Consistent with Chew et al.’s study, Queralto et al. (28) showed that 80% patients with idiopathic fecal incontinence showed a 60% mean improvement of their incontinence score after a month-treatment of TENS without surgically implanted electrode. The application of TENS to postoperative urinary retention after cesarean section has made some progress, and the commonly used modalities are low-frequency electrical stimulation and pulsatile smooth muscle stimulation (29). Clinical observations revealed that four-fifths of women receiving TENS demonstrated measurable voiding parameter enhancements, particularly in urinary stream intensity (30).

In recent years, more clinical experience has been accumulated in the application of TENS therapy to pelvic floor dysfunction than to other diseases, but the relationship between treatment parameters and efficacy of different types of pelvic floor dysfunction should be paid attention to in the future use (31).

### 2.3 Improvement on sexual dysfunction

TENS has also shown promising results in addressing postpartum sexual dysfunction, particularly dyspareunia (painful intercourse) and pelvic floor-related issues. Mechanistically, TENS targets pelvic floor hypertonicity and nerve sensitivity by delivering low-voltage electrical pulses to stimulate muscle relaxation and reduce pain perception. After 4 months of TENS treatment (15 min of 100 Hz frequency, a pulse width of 50 μs), Filippo Murina et al. (32) conducted a RCT and found that there was a 52.1% reduction in the dyspareunia VAS scores in women with provoked vestibulodynia. They also indicated that the female sexual functioning index (FSFI) measure also improved after TENS therapy. Zimmerman et al. (33) also demonstrated that TENS could significantly improve female sexual dysfunction symptoms. The average total FSFI score in these patients significantly increased either after six sessions, 12 sessions, or at study completion (*p* < 0.05 for all time points). Quantitative analysis revealed statistically significant improvements (*p* < 0.05) in FSFI scores across genitally-mediated arousal mechanisms, particularly in lubrication capacity, sexual arousal responsiveness, and orgasmic function, collectively reflecting enhanced genital neurovascular reactivity. Integrating patient-controlled TENS into multimodal therapeutic protocols produced clinically meaningful outcomes, with remarkable pain reduction and reduced vestibulectomy requirement. This sustained therapeutic persistence not only validates the clinical rationale for neuromodulation in refractory provoked vestibulodynia management, but also reinforces its pathophysiological reclassification within chronic pelvic pain syndromes mediated by central sensitization mechanisms. After TENS treatment, Marleen S Vallinga et al. (34) showed that sexual functioning scores on the FSFI questionnaire had improved significantly and these scores continued stable at follow-up. Furthermore, sexually-related personal distress scores had also improved dramatically after TENS treatment (*p* = 0.01). A previous study indicated that genital sensations during the preorgasmic and orgasmic phases in ReGS were conveyed via Adelta and C fibers, while being suppressed by Abeta fibers (35). The authors demonstrated that conventional TENS treatment is a promising therapy for women with restless genital syndrome. With observation of a significant improvement on the Marinoff dyspareunia scale and the FSFI, Murina et al. (36) found that TENS could serve as a straightforward, effective, and safe option for the three - month, short - term treatment in female sufferers with vestibulodynia. On the other hand, combining TENS with biofeedback and pelvic floor muscle training can enhance the sexual outcomes of these patients.

Pelvic floor exercise is one of the important ways for the improvement of sexual function in postnatal women. Hady unveiled a statistically significant disparity in Vaginal Laxity Questionnaire (VLQ) scores and pelvic floor muscle (PFM) strength between TENS and pelvic floor exercises, while TENS was markedly more effective in boosting PFM activity and improving VLQ scores among women with vaginal laxity. A previous RCT showed that both pelvic floor muscle training (PFMT) alone or in combination with TENS contributed to the improvement of female sexual dysfunction. These studies demonstrate that TENS has a comparable effect on sexual function or even superior effectiveness on improving the female sexual health.

TENS, especially when combined with biofeedback and pelvic floor rehabilitation, is a safe and effective intervention for postpartum sexual dysfunction. It addresses both physiological (muscle hypertonicity, nerve pain) and psychological (anxiety, depression) components, offering a holistic approach to postpartum recovery.

### 2.4 Improvement on mental stress and sleep disorders

Postpartum mental stress refers to the psychological pressure and strain that women may experience after giving birth (37). It encompasses a range of emotional and mental states, including feelings of anxiety, depression, irritability, and exhaustion (38). Postpartum mental and psychological health is also a key factor in determining the recovery process of patients. Female patients have a higher probability of developing psychosomatic disorders than male patients due to their physiological and psychological characteristics (39). Preoperative emotional disorders such as anxiety can produce large amounts of glucocorticoids through excessive activation of the hypothalamic–pituitary–adrenal axis, inhibiting the release of central analgesic substances and receptor function, thus leading to increased postoperative pain, the ineffectiveness of analgesic drugs and even the development of chronic pain (40). Postpartum mental stress can have a significant impact on a woman’s wellbeing and her ability to care for herself and her baby. It is important to recognize and address these issues promptly to ensure a healthy postpartum recovery.

Traditional Chinese medicine treatment has unique advantages in this regard, commonly used modalities include affective therapy, auricular pressure beans, acupuncture, etc. (41). TENS can offer potential benefits for postpartum mental stress management. A recent study showed that postpartum woman who received cranial electrical stimulation has a positive effect on improving the subjective anxiety state of postpartum status, to a certain extent, is conducive to reducing the patient’s postpartum pain and reduce the incidence of complications, while the hemodynamic parameters such as blood pressure, heart rate and so on, there is no significant effect (42). TENS may enhance endogenous opioid release (e.g., *β*-endorphins) and regulate *γ*-aminobutyric acid (GABA) activity, which are critical for mood stabilization and stress response. Zhang et al. (43) reported that the occurrence of potential postpartum depression at 3 days was 6.6% in the epidural analgesia group, and 2% in the TENS group (*p* = 0.04), indicating TENS significantly improved postpartum depression with reduced stress and enhanced recovery. In postpartum women with dyspareunia or pelvic floor dysfunction, TENS combined with pelvic floor exercises reduced pain-related anxiety and depressive symptoms, likely due to improved physical function and pain control. By alleviating pain and balancing autonomic activity, TENS may enhance sleep architecture, addressing sleep deprivation—a major contributor to postpartum mental stress. However, a previous study (44) indicated that TENS as an adjunct to usual care was not more effective than usual care alone in improving sleep quality in women with pregnancy.

Though effective in multiple aspects on postnatal women, TENS was found to be associated with potential autonomic disturbances (e.g., changes in heart rate variability, blood pressure fluctuations due to hormonal shifts and cardiovascular adaptations postpartum) and postnatal psychiatric disorders (e.g., anxiety, depression due to serotonin dysregulation in postpartum status). Therefore, it is recommended to monitor and mitigate the risks autonomic disorders (e.g., electrode placement, intensity adjustments) and emphasized the need for adjunctive therapies (e.g., cognitive-behavioral therapy) when TENS is used in psychiatric contexts.

It should be noted that the efficacy of TENS is affected by the psychological factors of the individual patient, so there may be greater variability in the time required for different diseases and patient groups, stimulation parameters, and how to form an individualized treatment plan will become an important direction for future research (45).

### 2.5 TENS on other female reproductive system disorders

Primary dysmenorrhea is a common gynecological disease, which refers to the cyclic lower abdominal cramping pain that occurs during or between menstruation in the absence of organic pathology, and seriously affects the patient’s work and life (46). In recent years, electrical stimulation therapy has been applied to the treatment of primary dysmenorrhea with a eligible result (47). The elevation of prostaglandins in the myometrium, local ischemia and hypoxia at the peripheral nerves increase the sensitivity of neurons to pain and other reasons will trigger dysmenorrhea symptoms (48). The researchers evaluated the therapeutic effect of TENS on primary dysmenorrhea in comparison with a placebo treatment group by sequentially applying two programs of electrical stimulation to the abdominal area above the pubic bone, namely, continuous high-frequency 100-Hz electrical stimulation and different frequencies of 60-Hz electrical stimulation in continuous and discontinuous modes (49). The electrical stimulation with different frequencies of 60, 80, and 100 Hz in continuous and discontinuous modes was observed to be superior to the placebo group in terms of therapeutic effect, patient satisfaction and onset of action at different time intervals. A study in China included both transcutaneous electrical acupoint stimulation (TEAS) and TENS to compare the differences in therapeutic effects between different types of electrical stimulation. The difference between the two groups was mainly in the location of the electrode pads, which were arranged on acupuncture points on the surface of the skin in the TEAS group, while the TENS group was arranged in the most obvious place of the pain and the arterial area. (Output frequency 100 Hz, pulse width 100 μs), and a 7-d pro-cyclic regimen (output frequency 10 Hz, pulse width 300 μs) before menstruation. It was observed that the pain scores and uterine artery blood flow parameters of the two groups were better than those of the control group, but the difference in near-term efficacy between the two groups was not statistically significant, but the long-term efficacy needs to be further studied (50).

Chronic pelvic pain (CPP) is a kind of disease with non-periodic chronic pain in the pelvic floor and related tissues as the main symptom, and the course of the disease is easy to be prolonged and recurring, and it usually lasts more than 6 months (51). A meta-analysis demonstrated that TENS application significantly reduced CPP in women with dysmenorrhea, while the TENS parameters should be set on the duration of at least 20 min, with 50–400 μs pulse duration and 2–120 Hz frequency (52). For patients with excessive pelvic floor muscle tension, biofeedback - assisted pelvic floor muscle training was implemented. Improvements in pain symptoms, pelvic floor EMG parameters, and muscle spasm were noted post - treatment, offering new diagnostic and treatment ideas for such patients (53).

Pelvic stasis syndrome is often characterized by chronic pelvic pain, accompanied by long-term varicose stasis of the pelvic veins. Previous treatment methods to drug therapy, surgery and interventional therapy, but the efficacy of the treatment is not long-lasting, traumatizing the patient and complications, and other shortcomings limit the scope of its application (54). A study randomly divided 80 cases of clearly diagnosed pelvic stasis syndrome into TENS treatment group and control group, the results showed that after TENS treatment, the internal diameter of the pelvic vein narrowed, hemodynamics accelerated, the patient’s self-awareness of the degree of pain is reduced, and the quality of life has been significantly improved (55). However, it is worth noting that some studies have shown that electrical stimulation therapy is contraindicated in thrombophilia because it may accelerate venous thrombosis. High-intensity TENS may induce localized muscle contractions, potentially exacerbating venous stasis (a risk factor for deep vein thrombosis). Also, electrical stimulation could theoretically promote endothelial activation or platelet aggregation in susceptible individuals. Hormonal shifts (e.g., elevated thrombin generation postpartum) and cesarean-related immobility compound thrombotic risks, necessitating heightened caution. However, few studies have focused on TENS and the risk of thrombophilia, indicating a great need to conduct some large-scale relevant studies on this issue.

Pelvic inflammatory diseases are the main cause of chronic pelvic pain, and if not diagnosed and treated in a timely manner, it will affect the quality of life of patients, and may even lead to pelvic abscess, infertility and other serious complications (56). A study combined Kangwu anti-inflammatory suppository with TENS to treat chronic pelvic pain caused by pelvic inflammatory diseases, and observed that patients’ symptoms and quality of life improved significantly after treatment (57). It was observed that patients’ symptoms and quality of life improved significantly after treatment, and the effect of the combined treatment was better than that of the two modalities alone, with no significant adverse reactions. The authors speculate that this therapeutic effect may be related to the stimulation of blood circulation by electric current, which causes a measurable increase in local skin temperature and accelerates the absorption of inflammatory substances (58).

The above studies implied that TENS is a promising complementary intervention for several postpartum disorders. Future research directions may focus on the following aspects, such as whether patients with different degrees of postnatal disorders should be treated with different parameters of electrical stimulation, the development of the treatment cycle and the long-term reproductive outcomes after TENS treatment in different patient subgroups.

## 3 Limitations and perspectives

Table 1 listed the characteristics and the main findings of the included studies aforementioned. Based on the above comprehensive review, TENS is a versatile tool in postpartum rehabilitation, addressing physical and psychological sequelae. However, several limitations hinder its widespread application and efficacy. First, although TENS reduces postpartum pain, pelvic floor dysfunction, long-term data are lacking. Second, mental and sexual health benefits may depend on continuous use, but adherence to long-term protocols is poorly documented. Third, optimal TENS parameters (frequency, pulse width) for postpartum conditions are inconsistently defined. Fourth, while TENS is generally safe, improper use (e.g., high-intensity currents) may cause skin irritation or interfere with pacemakers, thus postpartum populations with comorbidities (e.g., epilepsy) require cautious application. Fifth, as shown in Table 1, most of the included studies provided the different TENS parameters, assessment tools, and limited results to pool the OR with a 95%CI via a meta-analysis. While TENS offers a non-invasive option for postpartum recovery, its limitations, including inconsistent protocols, short-term focus, and understudied mental health applications, highlighting the need for larger trials, standardized guidelines, and integration into multidisciplinary care. Addressing these gaps could enhance its role in comprehensive postpartum management.

**Table 1:** Characteristics and the main findings of the included studies.

Study	Study area	Mean age (years)	Sample size	Spontaneous delivery n (%)	TENS parameters	Comparison	Main findings
Zhang (43) 2018	China	28.55 ± 2.50	51	46 (90.2%)	An intermittent pulse width of 100 μs and a frequency of 100 Hz	Doula	VAS pain scores in the TENS group decreased significantly compared to the doula group, while the occurrence of potential postpartum depression was 2% in the TENS and 1.3% in the doula group.
Zakariaee (15) 2019	Iran	39.3 ± 1.12	40	NA	60 min at a frequency of 100 Hz and 75 μSec pulse.	Normal control	A significant difference was observed between the mean pain severity of the TENS group and that of the group with walking activities (*p* = 0.04) and the mean pain severity of the lying position (*p* = 0.008).
Kasapoğlu (9) 2020	Turkey	30.6 ± 4.0	45	NA	100 Hz frequency with a pulse width of 75 μs, for 30-min sessions a day.	Normal control	There was a significant difference in the instant Visual Numeric Scale (VNS) scores for abdominal pain at 2, 6, 24, and 48 h in favor of TENS treatment (*p* < 0.05).
Deligiannidis (59) 2022	USA	33.7 ± 3.9	25	NA	Range 2.0–3.0 mA, using biphasic square waves with pulse width of 20 microseconds.	No control group	Transcutaneous auricular vagus nerve stimulation was well tolerated and served as an effective non-pharmacological treatment for major depressive disorder with peripartum onset.
Movahedi (60) 2022	Iran	30.10 ± 4.01	50	46 (92%)	An intensity of 10–18 mA and a frequency of 100 (Hz) for 30 min	Normal control	Labor pain was significantly less in TENS than in the control group (*p* < 0.001). There was no significant difference between the groups in terms of labor duration and maternal and fetal outcomes.
Velingkar (61) 2022	India	28.28 ± 3.98	25	NA	Continuous TENS of 100 Hz was applied for 30 min, one session per day.	Normal control	Administration of TENS shows an improvement in pain intensity and functional activities.
Silva (17) 2019	Spain	33	1	NA	A preset fixed frequency (125 Hz) and pulse duration (100 microseconds) for 30 min per session, 4 times a day.	No control group	TENS showed a good analgesia without any adverse effects for the mother or child.
Baransel (62) 2024	Turkey	≥26 (67.4%)	46	NA	100 Hz frequency with a pulse width of 75 μs, for 30-min sessions a day.	Normal control	There was a statistically significant difference in the instant Visual Numeric Scale (VNS) scores for abdominal pain at 2, 6, 24, and 48 h in TENS group (*p* < 0.05).
Pilch (11) 2024	Poland	32.0 ± 5.2	52	NA	A frequency of 100 Hz, with a pulse duration of 100 μs.	Normal control	TENS group had decreased pain intensity immediately after the intervention compared to the control group (*p* = 0.002). During the first stage of the verticalization, the smallest increase in pain was observed in the TENS (*p* < 0.001 compared to the control group).
Özen (12) 2024	Turkey	18–45	30	NA	A frequency reached 100 Hz, the pulse width reached 100 μs.	Normal control	Pain significantly decreased and comfort increased after having a cesarean delivery in TENS group compared to the control group.

TENS = transcutaneous electrical nerve stimulation; VAS = Visual Analogue Scale; NA = Not available.

## 4 Conclusion

In summary, TENS has been maturing, its practical value is gradually increasing, and it has been gradually applied to the rapid postnatal rehabilitation and common postpartum reproductive system-related diseases. TENS is a safe and effective adjunct therapy for postpartum disorders, addressing both physiological pain and indirect psychological stressors. There are some challenges in conducting postnatal tens studies, including heterogeneity of postnatal disorders, lack of standardized protocols, ethical considerations (e.g., informed consent, equity in access, and therapeutic misconception). In future, it should advocate for consensus guidelines on TENS parameters (e.g., frequency, duration) tailored to postnatal populations. Also, involving ethicists, obstetricians, and patient may facilitate in study design to safeguard participant autonomy and welfare. While further research is needed to standardize protocols and validate mental health benefits, its non-invasive nature and adaptability position it as a cornerstone of modern postpartum rehabilitation programs. Clinicians should prioritize patient education and multidisciplinary approaches to maximize recovery outcomes.
